# Salient Arithmetic Data Extraction from Brain Activity via an Improved Deep Network

**DOI:** 10.3390/s23239351

**Published:** 2023-11-23

**Authors:** Nastaran Khaleghi, Shaghayegh Hashemi, Sevda Zafarmandi Ardabili, Sobhan Sheykhivand, Sebelan Danishvar

**Affiliations:** 1 Department of Electrical and Computer Engineering, University of Tabriz, Tabriz 51666-16471, Iran; khaleghi.nstr@tabrizu.ac.ir; 2 Department of Computer Science and Engineering, Shahid Beheshti University, Tehran 19839-69411, Iran; sh.hashemi@alumni.sbu.ac.ir; 3 Electrical and Computer Engineering Department, Southern Methodist University, Dallas, TX 75205, USA; szafarmandiardabilii@smu.edu; 4 Department of Biomedical Engineering, University of Bonab, Bonab 55517-61167, Iran; s.sheykhivand@tabrizu.ac.ir; 5 College of Engineering, Design and Physical Sciences, Brunel University London, Uxbridge UB8 3PH, UK

**Keywords:** arithmetic content, visual perception, electroencephalogram, deep learning, MNIST

## Abstract

Interpretation of neural activity in response to stimulations received from the surrounding environment is necessary to realize automatic brain decoding. Analyzing the brain recordings corresponding to visual stimulation helps to infer the effects of perception occurring by vision on brain activity. In this paper, the impact of arithmetic concepts on vision-related brain records has been considered and an efficient convolutional neural network-based generative adversarial network (CNN-GAN) is proposed to map the electroencephalogram (EEG) to salient parts of the image stimuli. The first part of the proposed network consists of depth-wise one-dimensional convolution layers to classify the brain signals into 10 different categories according to Modified National Institute of Standards and Technology (MNIST) image digits. The output of the CNN part is fed forward to a fine-tuned GAN in the proposed model. The performance of the proposed CNN part is evaluated via the visually provoked 14-channel MindBigData recorded by David Vivancos, corresponding to images of 10 digits. An average accuracy of 95.4% is obtained for the CNN part for classification. The performance of the proposed CNN-GAN is evaluated based on saliency metrics of SSIM and CC equal to 92.9% and 97.28%, respectively. Furthermore, the EEG-based reconstruction of MNIST digits is accomplished by transferring and tuning the improved CNN-GAN’s trained weights.

## 1. Introduction

Neural activity decoding is of great importance in neurocognitive research. Electroencephalography is a popular technique for recording brain activities, and analyzing EEG results in response to unique stimulation is the objective of brain–computer interface (BCI) applications. An essential part of the human perception of the surrounding environment occurs via vision, and inferring the connection between brain response and vision has been considered in several neuroscience studies. Patterns related to brain signals can be distinguished, corresponding to various classes of visual stimuli [1,2,3].

Classifying electroencephalogram (EEG) recordings is the central part of neural decoding, and various methods have been presented for this purpose [4,5,6]. In recent years, the deep learning approach has attracted the attention of researchers [7,8], and deep network applications have been extended to the concept of EEG signal processing in BCI for emotion recognition [9], fatigue and sleep databases [10], epilepsy diagnosis and treatment [11], motor movement/imagination databases [12], and SSVEP datasets [13]. Deep learning models have also been employed to explore the effect of visual stimulation on brain activity. For example, sequential LSTMs were used by Simone Palazzo et al. in [14] to realize the classification of the EEG-ImageNet signals corresponding to the brain activity of human volunteers when displaying a set of images from the ImageNet dataset. In another study [11], the use of deep network structure has been considered for image reconstruction from brain signals.

The hemispheric lateralization concept of the brain attracted the attention of Fares et al. [15], who used the information of hemispheric regions to construct a two-directional deep neural network for classifying the EEG-ImageNet signals. Nicolae Cudlenco et al. in [16] considered the Gabor filtering of EEG records to extract hidden features related to visual concepts in EEG signals. The projection of the entire spectrum on the space of Gabor wavelets was considered across a relatively large range of frequency bands to extract discriminative features, and they compared the performance of classification using ridge regression and deep network approaches. The processing of changes in the shape and color of objects was explored in a study by Nalin Mathur [17]. EEG data were collected using a feature-binding experiment that required subjects to detect changes in color and shape binding after 100 ms and after 1500 ms. The experiment was implemented to predict two stages of color and shape processing of visual stimuli in brain EEG data recordings. Familiar and unfamiliar face detection was considered by Lidia Ghosh et al. [18]. A deep model has been designed by them to quantify the ability of face perception in human subjects by classifying the EEG responses to facial images.

The brain response to natural visual stimulation was investigated by Ghebreab et al. [19] and was predicted using EEG responses. Similar research was carried out by Kay et al. in 2008 [20], but a better accuracy was achieved in 2010 [19]. Visual stimulation effects of the orientation of images [21], position of objects [22], and color of things [23] on brain activity have been explored in other studies.

The salient points in a picture have an essential effect on the visual interpretation occurring in the brain. Ulman and Koch [24], in 1985, proposed the concept of the optical salient region in an image to identify a region’s dominance in the brain’s visual information processing. Another model designed to improve understanding of the salient map was introduced by Itti et al. [25] in 1998. According to this work, a scene’s uniqueness, distinctiveness, and rarity are essential parts of saliency detection. Many visual saliency detection models have been developed following the model proposed by Itti [25].

Understanding how the salient region influences brain signals is vital in eliciting the visual system’s function. Although some works have been dedicated to the connection between the saliency of visual stimulation and brain activity, the relationship between salient arithmetic content and brain activity has yet to be studied. Furthermore, the use of time-related information of EEG signals has yet to be considered to extract the connection between visually evoked brain activity and salient arithmetic content.

The classification step is the critical step to achieve the salient arithmetic content corresponding to the visual stimuli. This work introduces a deep convolutional network to recognize the arithmetic category of EEG records. The proposed network consists of a convolutional network and a generative adversarial network to extract the arithmetic visual saliency map related to the recorded EEG signals. The overall model realizes the extraction of salient arithmetic data through visually evoked EEG records.

The achievements in this article can be summarized as follows:

(i) Presentation of an effective deep network to acquire salient arithmetic content of visual stimulation from EEG recordings.
(ii) Extraction of the arithmetic data is possible using the proposed architecture.
(iii) Presentation of a convolutional deep network for extracting EEG features to recognize 10 patterns of EEG recordings corresponding to 10-digit categories.
(iv) A 14-channel time sample of the EEG dataset is imposed directly as an input signal to the proposed CNN-GAN. The removal of feature vector extraction step results in decreasing the computational load.
(v) It paves the way to connect three modalities: image data, visual salient data, and EEG signals.

The remaining parts of this article are arranged as follows. Related works to the saliency models have been explained in Section 2. The details of the MindBigData dataset and the mathematical precursors related to convolution, GAN, and saliency metrics are explained in Section 3. Section 4 describes the architectural and structural details of the proposed EEG-related arithmetic data recognition framework. The investigational results and validation against other newest methods are provided in Section 5. The conclusions are in Section 6.

## 2. Related Work

In early studies about salient content recognition [26,27], the saliency calculation was performed with pixel contrast. Hu et al. [28], in 2005, considered the geometric features of different regions and used principal component analysis to estimate the salient subspaces. A saliency detection method has been proposed with some simple operations of binarization, threshold decomposing, and edge detection for every pixel by Rosin et al. [29]. Estimating the salient content has been considered with isocenter clustering and curvedness by Valenti et al. [30].

Visual attention procedure has been used for modeling the saliency recognition. This procedure is a selective phenomenon for training the brain to understand the surrounding environment. Bottom-up pre-attentive feature-based and attentive task-based top-down processes have been suggested by Neisser [31] in 1967.

Bottom-up models use visual features of orientation, color, and intensity. Zhang et al. in 2013 [32] and Mauthner et al. in 2015 have proposed some saliency likelihood calculations for this type of modeling. Top-down saliency models consider the primary knowledge about features and the intentions corresponding to the brain task. The 2011 model by Zhao et al. [33], the work by Xu et al. [34] in 2014, and the model by Yang in 2017 [35] are examples of a top-down saliency process. Another category is based on the combination of top-down and bottom-up approaches. In these models, the top-down approach helps implement the given task, and the bottom-up technique detects the salient points.

Many neural networks have been proposed to implement models corresponding to saliency detection processes. Shengfeng He et al. [36], in their recent work, have assessed the convolutional neural networks to detect the salient object. The efficiency of these neural networks in salient object recognition has been analyzed by Ghuanbin et al. [37]. A one-dimensional convolutional neural network has been proposed by He et al. [36] to solve the saliency problem. Also, features of decomposed regions corresponding to different input scales have been imposed to MLP with two dense layers in a work by Yu and Li [37] in order to obtain a binary label for salient region detection.

Salicon [38] is one of the recent methods based on deep neural networks proposed for saliency detection. Transfer learning of GoogleNet, VGG-16, and AlexNet has been used in this model such that the weights of this model have been initialized with transfer learning. Another recently proposed deep network for saliency modeling is SalNet [39]. This network consists of two shallow and deep networks. The shallow network contains five convolutional and dense layers. A total of 10 layers have been employed in the deep part of the SalNet. More than 25 million parameters are trained during the deep learning process of the network.

Some recent studies have been dedicated to realize the connection between the salient content and brain activity. A model has been presented by Humbeeck et al. [40] to evaluate the effect of visual saliency on the amplitude of the EEG recordings. Fixation positions of the pupil in the eye via the eye-tracker and the brain activity using the EEG device have been considered in the modeling. The study by Zhen Liang et al. [41] in 2018 presented an approach based on the model by Tavakoli et al. [42] that shows this interaction with the use of video stimuli. A good accuracy has been reported in [41] in reconstructing and predicting the temporal distributions of the features corresponding to salient regions using the extracted features of EEG recordings. In robot navigation and object identification in recorded images by robots by Mao et al. [43], P300 waves of the volunteers have been considered in identifying the objects of interest. A siamese network has been proposed in [44] through multimodal learning of image and EEG modalities for visual saliency detection. A cost function has been defined in the modeling, and its maximization has been considered to realize the connectedness of the modalities to the salient regions. Different scales of image masks have been considered for calculating the cost function and estimating the visual saliency. The mapping between EEG patterns and the salient picture of the visual stimulation has been realized in [45]. A deep network using a graph-embedded representation of EEG recordings has been introduced to acquire this map. Functional connectivity in different brain regions has been considered in exploring the interaction between visual saliency and brain activity [46]. 

This article proposes a novel approach for extracting the salient arithmetic content from visually evoked 14 EEG signals. In the next section, we explain the details of the database setting and the mathematical preliminaries to implement the proposed method.

## 3. Materials and Methods

The MindBigData [47] is used in this article, and recording details are explained in this section. The mathematical precursors of convolution 1-d, dropout, dense, and batch normalization layers will be presented. Furthermore, the details of generative adversarial networks, in addition to saliency metrics, are described.

### 3.1. Database Settings

**Figure 1:**
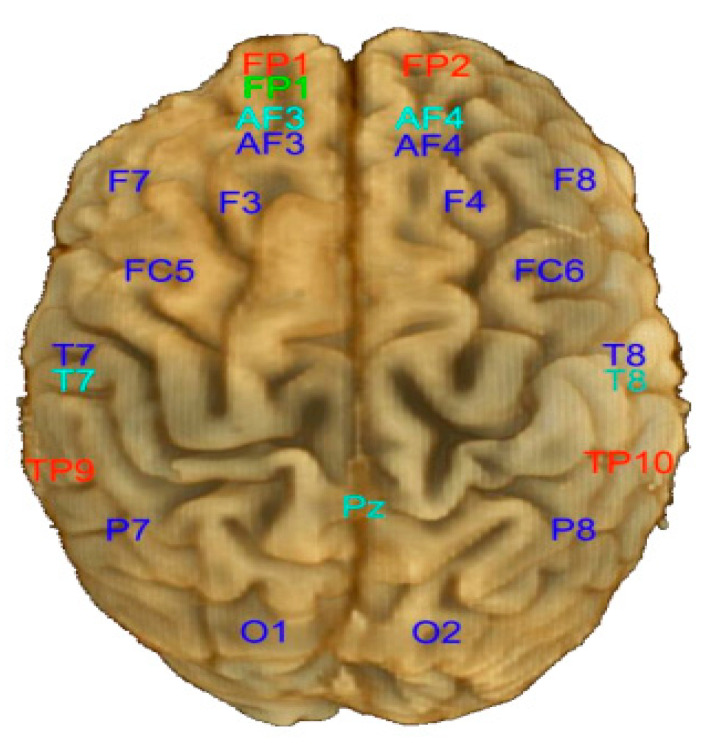
The placement of EEG electrodes of Emotiv EPOC.

The MindBigData has been recorded by David Vivancos [47] with the Emotiv EPOC device. This dataset has been gathered using a 14-channel cap. The placement of the EEG channel electrodes is shown in Figure 1, and the channel names considered in the recording procedure are A.F.3, F.7, F.3, FC.5, T.7, P7, O.1 in the left hemisphere and A.F.4, F.8, F.4, F.C.6, T.8, P8, O.2 in the right hemisphere as shown in dark blue in this figure. The channel names with details are described in Table 1.

**Figure 2:**
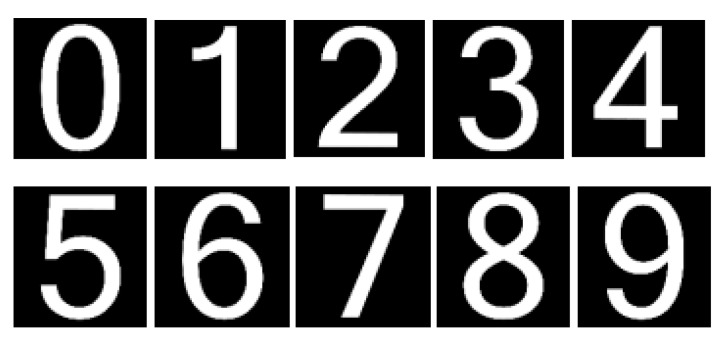
The digits of the MNIST dataset.

A total of 9120 brain signals of 2 s captured at a theoretical sampling rate of about 128 samples per second or 128 Hz are selected as MindBig dataset used in this paper. Total number of samples in each channel used for processing is 250. The brain signals were captured while a single digit from 0 to 9 corresponding to digits of the Modified National Institute of Standards and Technology (MNIST) dataset in Figure 2 has been shown for 2 s. The numbers have been represented on a 65-inch TV screen in a white font over a total black background. The appearance of digits was random, with a black screen between them. The number of 9120 EEG records corresponding to 912 signals of each category are considered for classification and salient arithmetic data extraction.

### 3.2. The Layers of Convolutional Neural Networks

The work of Hubel and Wiesel [48] can be considered the most important neural network in machine vision. The origin of this network goes back to the biological experiment conducted by them in 1962. The research conducted by these two individuals led to the discovery of simple and compound cells of vision. The identification of vision patterns was possible based on this study using these two types of cells. Simple cells in the vision system are responsible for detecting edges and columns in an image’s specific direction and location. In contrast, the detection capability of composite cells is not limited to a particular area of the image, and this is possible at any point of the picture. This composite cell capability is obtained by collecting information from several simple cells. The first convolutional neural network was proposed based on the concepts of simple and compound vision cells and designed in 1979 by Kunihiko Fukushima [49]. The first project using these networks was identifying handwritten figures of digits, which was carried out by Yan LeCun et al. [50,51], and satisfactory results were obtained in the study.

Each deep convolutional network is composed of several layers. The pooling layer is an essential layer in CNN, which minimizes the spatial size of feature vector maps obtained from the convolutional layer. This layer has no training parameter and performs a simple sampling. The most famous pooling layers are called average pooling and max pooling. For example, for maximum integration, a predefined window is considered that moves over the image to select the maximum value and ignore the rest of the numbers. The size of the filter and the size of the stride step in this layer are considered proportional to the optimal size for mapping the obtained feature of each layer.

The fully connected layer forms the final layer of CNN networks, which is used to classify the extracted feature maps. This layer is similarly present in multilayer perceptron (MLP) networks. After displaying the feature vectors obtained from convolutional layers, weight vector coefficients are assigned in this layer. The output corresponding to the number of classes available for classification can be achieved.

The following describes some other commonly used layers in CNN networks, including the random elimination layer and the batch normalizer layer. The use of the dropout layer in CNN networks strongly prevents the phenomenon of data overfitting in the training process. The function of this layer is to omit some neurons during training randomly. To optimize the coefficients, these randomly selected neurons are not considered during the learning process. Mathematically, neurons are discarded with probability (p-1), and other neurons are retained with probability (p).

The normalization layer is used to normalize the data inside the network. By performing various calculations on data, the distribution of data will change. The batch normalizer layer increases the training speed of the network by reducing the internal covariance of data distribution and accelerates the convergence process. The performance of this layer will be based on the calculation of the average and variance of data according to (1).

(1) $$\begin{array}{l} \mu_{B}=\frac{1}{n}\sum_{i=1}^{n}y_{i}^{l}\\ \sigma_{B}^{2}=\frac{1}{n}\sum_{i=1}^{n}{\left(y_{i}^{l}-\mu_{B}\right)}^{2}\\ {\hat{y}}^{l}=\frac{y^{l}-\mu_{B}}{\sqrt{\left(\sigma_{B}^{2}+\varepsilon \right)}} \end{array}$$

### 3.3. Generative Adversarial Networks

The generative adversarial networks (GANs) [7] include generator and discriminator networks. The generative model G consists of some layers to fit a random vector *y* with probability distribution *P(y)* into a desired data distribution. The discriminative part, named *D*, compares data from the expected distribution and data obtained from the generator part. These two networks are trained simultaneously, and the training will continue to see no improvement in network optimization. The cost function of GAN can be described as follows:

(2) $$\underset{Gen}{min}\underset{Disc}{max}V\left(D,\;G\right)=\underset{Gen}{min}\underset{Disc}{max}\;\left[E_{X\;P_{{}^{data^{\left(x\right)}}}}\left[logDisc\left(x\right)\right]+E_{Y\;p_{y^{\left(y\right)}}}\left[log\left(1-Disc\left(Gen\left(y\right)\right)\right)\right]\;\right]\;$$

In this loss e function corresponding to GAN, the desired data are depicted as *x*, and the random feature vector employed as input to the generator is represented with y. The generator’s output is represented by Gen(*y*), and the work of the discriminator is shown with Disc(*x*). The convergence will occur when the loss function according to each network is as close as possible to 1. The output of the discriminator is represented by Disc(Gen(*y*)). The probability distribution of generated data and the desired data is represented respectively, with $P_{{}^{data}}(x)$ and $p_{y}(y)$.

### 3.4. Evaluation Metrics for Classification and Salient Image Extraction

This section provides a brief description of classification and saliency evaluation metrics. The saliency feature map and the ground truth feature vector are two necessary inputs for calculating the saliency evaluation metrics. The level of similarity can be represented by considering these metrics.

The most used metrics for classification are described in (3) as sensitivity, accuracy, precision, and recall based on true positive (TP), true negative (TN), false positive (FP), and false negative (FN).

$$\mathrm{Sensivity}=\mathrm{Recall}=\frac{\mathrm{TP}}{\mathrm{FN}+\mathrm{TP}}$$

$$\mathrm{Accuracy}=\frac{\mathrm{TP}+\mathrm{TN}}{\mathrm{TP}+\mathrm{TN}+\mathrm{FP}+\mathrm{FN}}$$

$$\mathrm{precision}=\frac{\mathrm{TP}}{\mathrm{FP}+\mathrm{TP}}$$

(3) $$\;F1-score=\frac{\;precision+recall}{2}$$

Cohen’s Kappa coefficient is another metric for classification, as described in (4).

(4) $$\;kappa=\frac{2\times (TP\times TN-FN\times FP)}{\left(TP+FP)\times (FP+TN)+(TP+FN)\times (FN+TN\right)}$$

Three important metrics for evaluating saliency are described as follows.

Similarity metric (SIM) is used to measure similarity between distributions [52]. Normalization of the input signal vectors is performed, and the sum of minimum values at each pixel results as S.I.M. The saliency map is shown with S.M and the fixation map is represented with F.M:

SIM(SM, FM) = Σj min(SMj, FMj)   where     Σj SMj = Σj (FMj) = 1(5)

In (5), pixel locations are represented with j. The value of SIM is equal to one for inputs with identical distributions, while this metric would be zero if there is no similarity and overlap between distributions.

Another saliency evaluation metric is structural similarity (SS.I.M), calculated using different windows of an image [53]. The SSIM is computed considering two sampling windows, *m* and *n*, with size L × L:

(6) $$SSIM\left(m,\;n\right)=\;\frac{\left(2\times \mu_{m}\mu_{n}+k_{1}\right)\left(2\times \sigma_{mn}+k_{2}\right)}{\left(\mu_{m}{}^{2}+\mu_{n}{}^{2}+k_{1}\right)(\sigma_{m}{}^{2}+\sigma_{n}{}^{2}+k_{2})}$$

${µ}_{m}$: the average of *m*; $\sigma_{m}{}^{2}$: the variance for *m*;
${µ}_{n}$: the average of *n*; $\sigma_{n}{}^{2}$: the variance for *n*;
$\sigma_{mn}$: the covariance between *m* and *n*;
$k_{1}=\;{\left((d_{1})K\right)}^{2}$; $k_{2}=\;{\left((d_{2})K\right)}^{2}$;
K: the variation range of the pixel-intensities $\left(2^{\left(bits\;per\;pixel\right)}-1\right)$;
$d_{1}=0.01$ and $d_{2}=0.03$.

A metric for assessing the affine connectedness of distributions is Pearso.n’s correlation coefficient (CC) [52]. CC can be computed as in Equation (7).

CC(FM, SM) = σ(FM, SM)/σ(FM) × σ(SM)(7)

The covariance between FM and SM in (7) is represented with σ(SM, FM). This metric is unaffected by linear transformations. This evaluation metric would treat false negatives and false positives equally and, therefore, is a symmetric function. The similar magnitudes of the saliency map and the reference ground truth would result in high positive values of CC.

## 4. Proposed Convolutional Neural Network-Based Generative Adversarial Network

The details of the proposed convolutional neural network (CNN) for automatic visual arithmetic content identification from EEG signals and the proposed convolutional neural network-based generative adversarial network (CNN-GAN) for arithmetic data extraction from brain activity are elucidated in this section.

### 4.1. The Proposed Network Architecture

**Figure 3:**
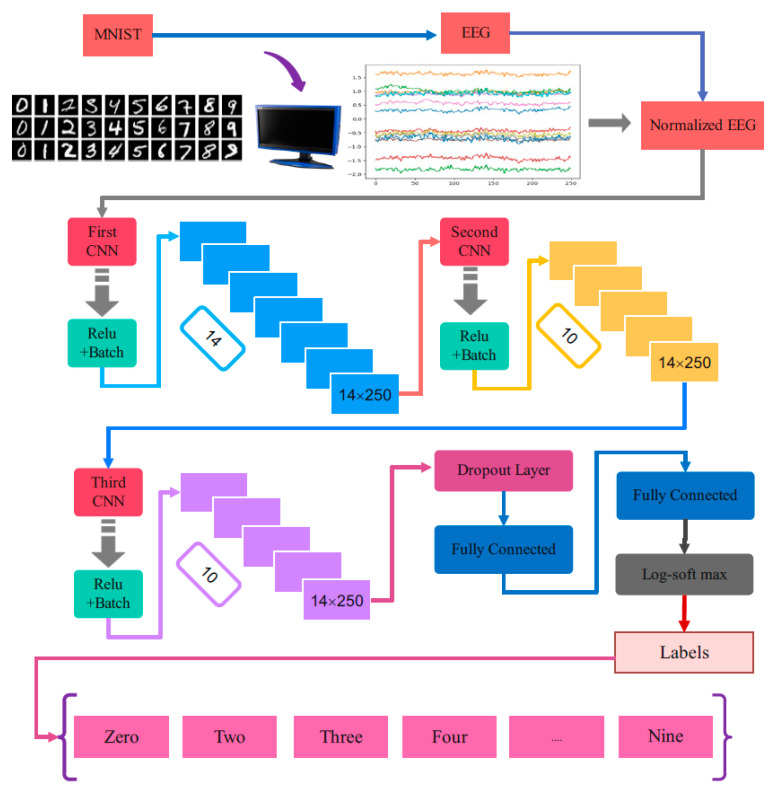
The block diagram of the layers in proposed CNN.

Figure 3 represents the schematic of the CNN fragment to classify the input EEG signal into the correct category of the MNIST dataset. The visual stimulation related to the MNIST dataset appears on an LCD to a human volunteer, corresponding to the considered timing of occurrence for each image and time-lapse between sequential images. The EEG signals are recorded during the experiment. Normalizing the EEG time samples is performed considering each EEG channel’s mean and standard deviation. The pre-processed EEG signals are applied as input to the proposed one-dimensional CNN network.

The structure of the CNN fragment of the network consists of three convolutional layers, as illustrated in Figure 3. The rectified linear unit is selected for the activation function in each layer. After each convolutional layer, a dropout and batch normalization are considered to prevent overfitting. Flattening of the output of the third convolutional layer is achieved. It is imposed to a linear layer, and the output vector of a dense, full-connected layer is passed through a log_softmax classifier layer to classify the input EEG signal. The output of the linear layer is a vector with 2500 elements, and it is the vector applieded as input to the next deep network of the proposed CNN-GAN to extract and obtain the MNIST images used as the stimulation. The details of the CNN network of the proposed architecture are explained in Table 2.

**Figure 4:**
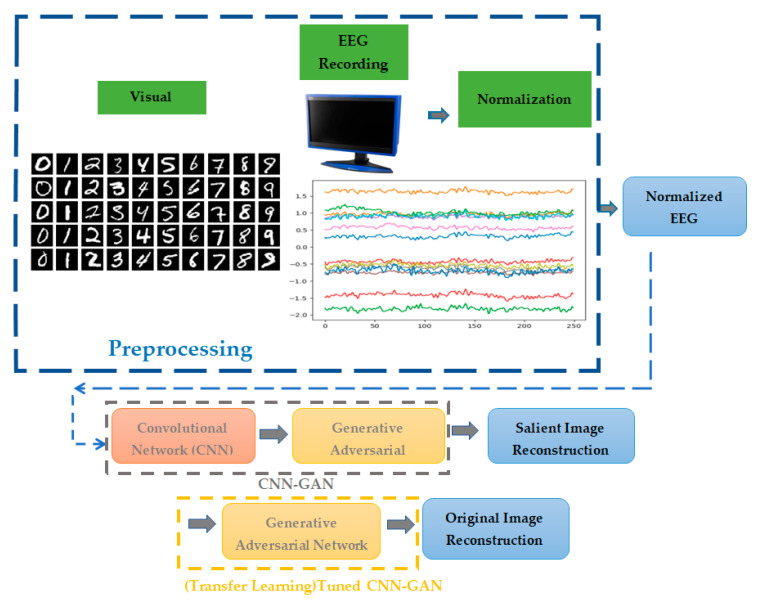
Details of the reconstruction procedure.

The proposed CNN-GAN consists of two fragments of sequential layers, as in Figure 4. The one-dimensional convolutional layers in the first part of the proposed network classify the EEG signals related to 10 different numbers of MNIST images with arithmetic content. EEG signal classification happens in this stage, and the signal can be classified according to the extracted features for 10 categories. After the preprocessing stage of the recorded EEG signals in response to the visual image with arithmetic content, we have several deep layers to extract the output vector of the first part of the proposed network to be applied to the next generator adversarial network. The second part consists of generator and discriminative networks in order to map the one-dimensional extracted feature vector of the EEG signal in the first part to the two-dimensional image array. The main layers of GAN in the proposed technique are two-dimensional convolutional blocks to reconstruct the salient images.

The salient images are created using the SALICON approach to apply as the ground reference data of the GAN network. The generative adversarial network is trained, and the network weights will be determined. After this stage of the procedure, tuning the weights of the trained network will be performed and transfer learning will be employed to reconstruct the original visual image stimulation.

**Figure 5:**
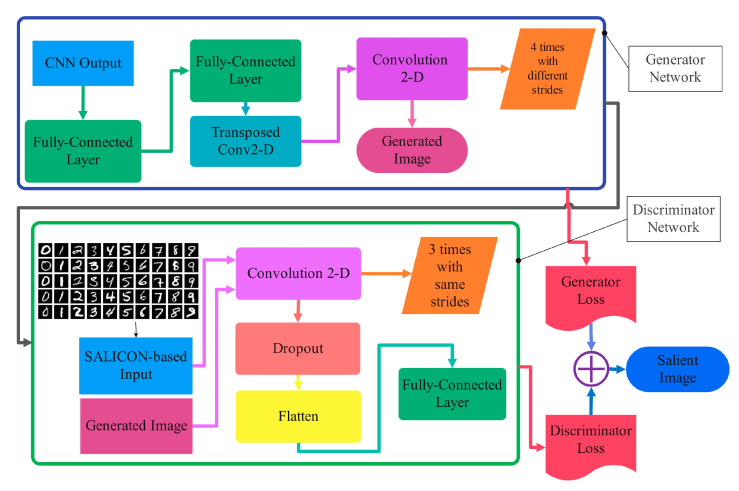
The details of the proposed CNN-GAN for visual salient image extraction.

The architectural details of the generator and adversarial networks can be seen in Figure 5. The flattened vector with 2500 elements is passed through a dense layer of the generator network and four transposed two-dimensional convolution layers. Also, an additional two-dimensional convolutional layer is needed to fit the output image dimension to the desired dimension. The adversarial consists of three sequential layers of two-dimensional convolutional layers. The output of these layers is imposed to the dropout layer, and then the flattened output vector passes a fully connected layer to judge about fake or real data.

**Figure 6:**
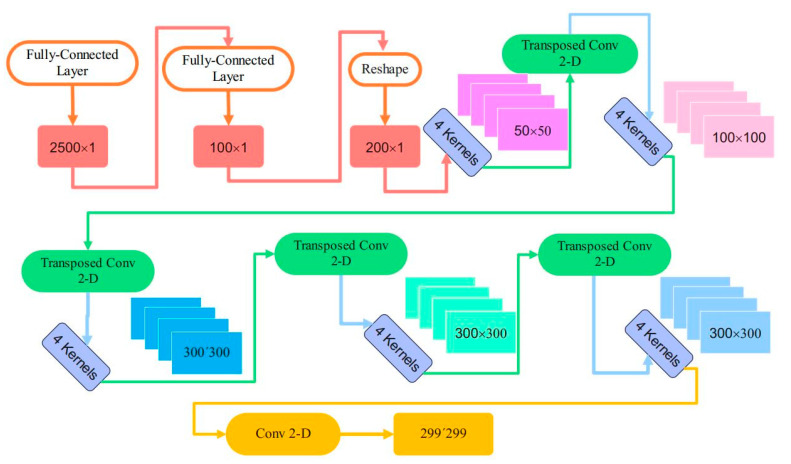
The output shape of layers in generator network of the CNN-GAN.

The structural and dimensional details of layers in the generator are represented in Figure 6 and Table 3. The input vector dimension is equal to 2500, and after passing through two dense layers, the output vector dimension is equal to 20,000. The reshaped vector of dense layer output is applied in the first transposed convolution layer. The number of kernels in transposed convolutional layers is considered equal to four to have four two-dimensional outcomes in each layer. Considering different kernels and strides in five transposed convolution layers according to Table 3, the dimensions of two-dimensional outputs are equal to 50 × 50, 100 × 100, and 300 × 300, as illustrated in this table.

**Figure 7:**
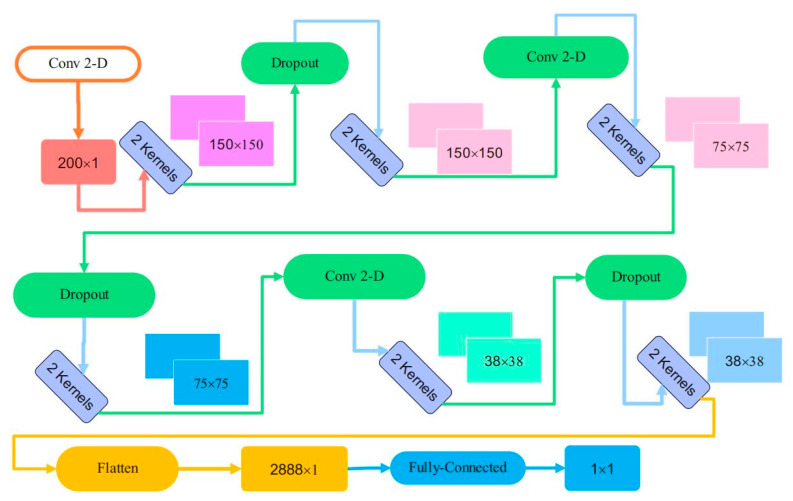
The output shape of layers in discriminator network of the CNN-GAN.

Table 4 and Figure 7 describe the convolutional layers’ structural details in the proposed framework’s discriminator part. Three convolutional layers with kernel size of 4 and stride length of 2 have been considered. Two filters have been applied to construct the output of each convolutional layer. Furthermore, the total number of parameters is illustrated in Table 4. Flattening of the last convolutional layer is performed to apply to the final dense full-connected layer.

**Figure 8:**
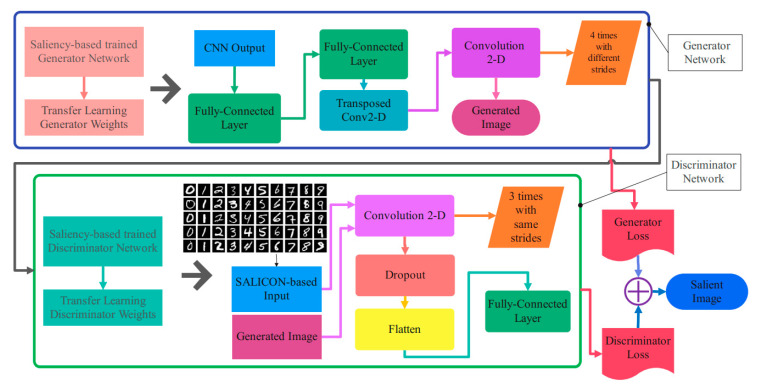
The details of transfer learning for image reconstruction.

The procedure to create the original image from brain activity using the salient image extraction deep network is illustrated in Figure 8. Transferring the network’s trained parameters for the visual stimulation’s salient image to the proposed architecture in this figure would result in the original image of visual stimulation. The initialization of the weights is performed by transferring the weights, and tuning of the weights is performed through cross-validation to acquire the original image.

### 4.2. Training and Evaluation

The training procedure is accomplished through cross-validation to adjust the network weights of the proposed CNN to the MindBig dataset. The trained parameters of this part are transferred to the proposed CNN-GAN to extract the salient images of visual stimulation from EEG recordings. Cross-entropy is utilized as a loss function for the training phase of CNN, and binary cross-entropy is employed for the training phase of CNN-GAN. Different parameters have been used through a trial–error technique to find the optimal values of the proposed architecture. Table 5 represents the values as search scope for the optimizer, cost function, and learning rate for the CNN and generative adversarial parts. The corresponding optimal values are obtained with trial and error and illustrated in this table.

## 5. Results and Discussion

The practical implementation results of the proposed CNN-GAN are discussed in this section. A laptop with a GTX 1050 GPU, 16 GB RAM, and a Core i7 2.8 GHz CPU is employed to implement the proposed framework for classification. Furthermore, the reconstruction procedure is accomplished with Python programming in the Google Colaboratory platform with fast GPUs.

**Figure 9:**
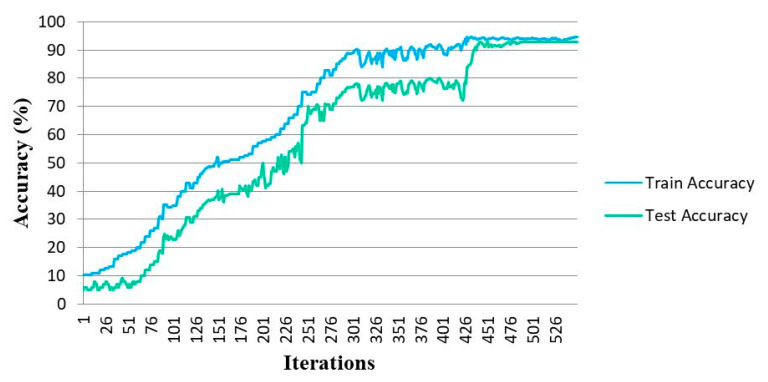
Train and test accuracy plots in CNN.

**Figure 10:**
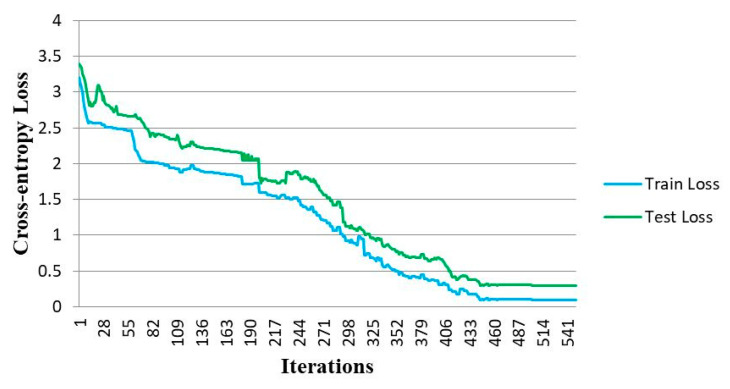
Train and test loss plots in CNN.

The train and test accuracy plots of the proposed CNN to classify the MindBig dataset into 10 categories of visually evoked brain signals corresponding to the numbers between zero and nine are represented in Figure 9. The train and test loss plots of the proposed convolutional network are illustrated in Figure 10.

The accuracy and loss plots are tracked for 530 number of iterations. The convergence of training and testing of the proposed CNN through the implemented 10-fold cross-validation is achieved after 420 iterations and the convergence of the CNN training for the classification of the MindBig dataset is acquired.

Furthermore, the proposed CNN’s efficiency compared to the other state-of-the-art deep networks is assessed with classification metrics including accuracy, Cohen’s Kappa coefficient, F1-score, and precision. The results corresponding to the evaluation metrics for LSTM [14], GNN [46], and CNN-LSTM are demonstrated in Table 6. A different number of layers has been considered for LSTM as the search scope, and three layers with 84.3% accuracy have been reported in this table. Four layers, as chebconv and graph convolutional layers, have been selected to report the graph neural network (GNN) performance for the MindBig dataset.

In addition to LSTM and GNN, a combination of three LSTM layers with two convolutional layers is considered and analyzed. This table confirms the efficiency of the proposed CNN for the classification of the MindBig dataset.

**Figure 11:**
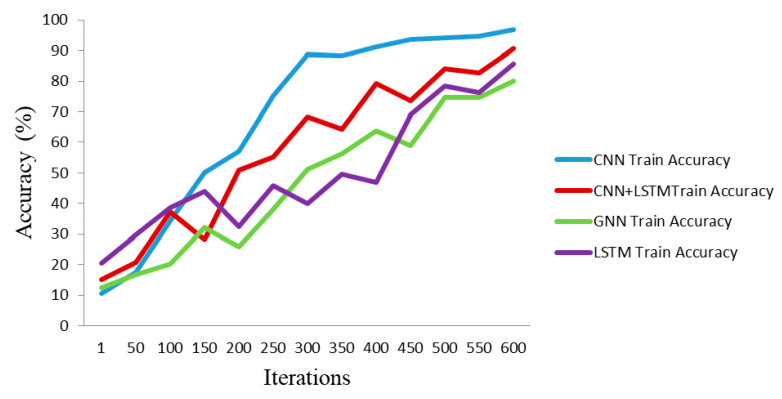
Comparison of train accuracy trends with state-of-the-art methods.

**Figure 12:**
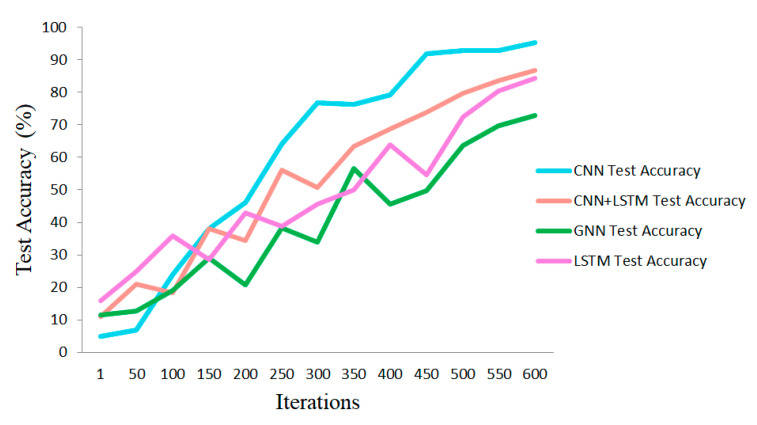
Comparison of test accuracy trends with state-of-the-art methods.

The accuracy trend plots of train and test procedures in each state-of-the-art method for MindBig dataset are illustrated in Figure 11 and Figure 12, respectively. As illustrated in these two figures, the efficiency of the proposed method against other state-of-the-art methods for classifying the MindBig dataset is observable.

**Figure 13:**
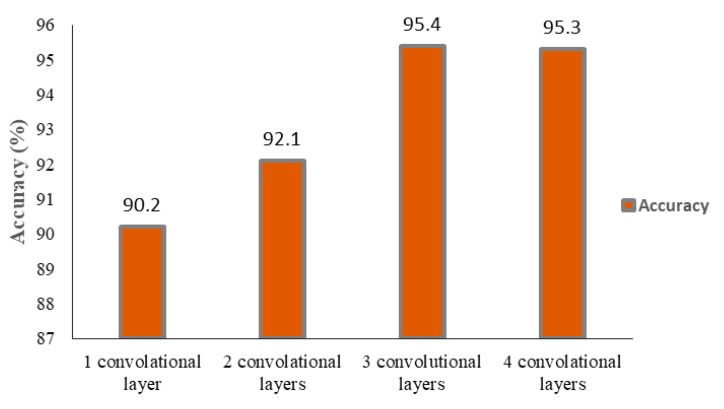
Training accuracy for a different number of convolutional layers in CNN.

**Figure 14:**
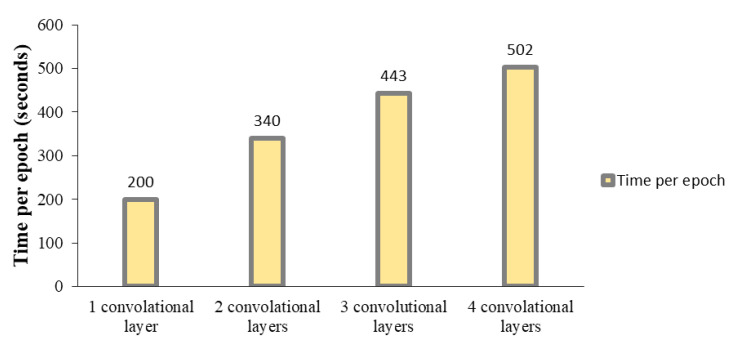
Processing time per epoch for different numbers of convolutional layers in CNN.

The effect of altering the number of convolution layers in performance and processing time in the training procedure is shown in Figure 13 and Figure 14. As can be seen, three convolution layers result in a desirable compromise between the accuracy and processing time of the proposed CNN.

**Figure 15:**
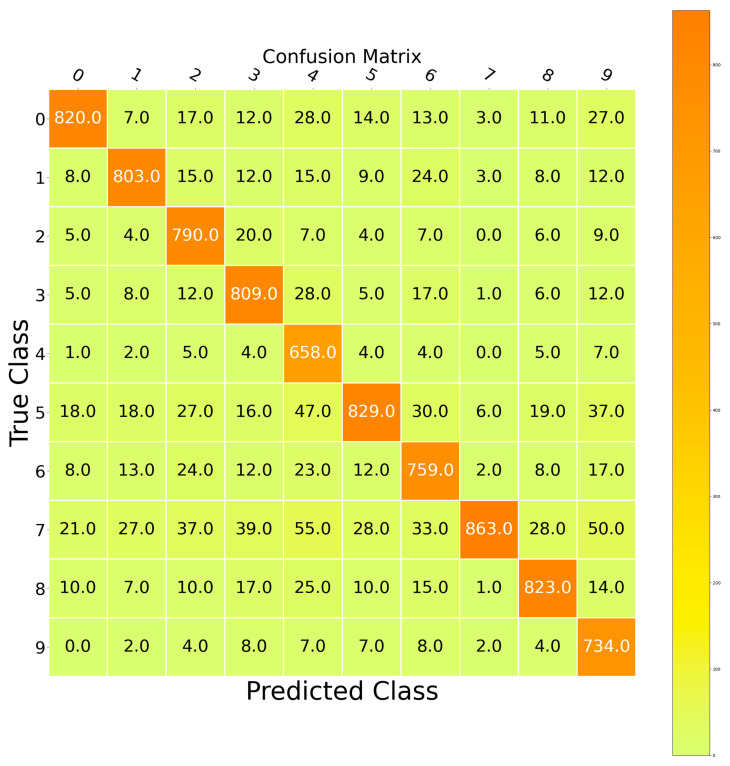
Confusion matrix of CNN.

Another way to assess the performance of the proposed architecture is the representation of the confusion matrix. Figure 15 exhibits the corresponding matrix of the proposed CNN to classify the MindBig dataset. This matrix confirms the efficiency of the proposed CNN.

The MindBig dataset consists of 9120 14-channel EEG signals. We can generate a set of signals through training a generative adversarial network (GAN) and add the generated signals to the base dataset to evaluate the performance of the proposed CNN. We generate 50 sets of 10 14-channel EEG signals according to different categories and add these 500 generated signals to the MindBigData. The generator part of the GAN consists of three transposed convolution 2-D layers, and the discriminator part of the GAN includes three convolution 2-D layers. The details of layers are presented in Table 5 and Table 6.

**Figure 16:**
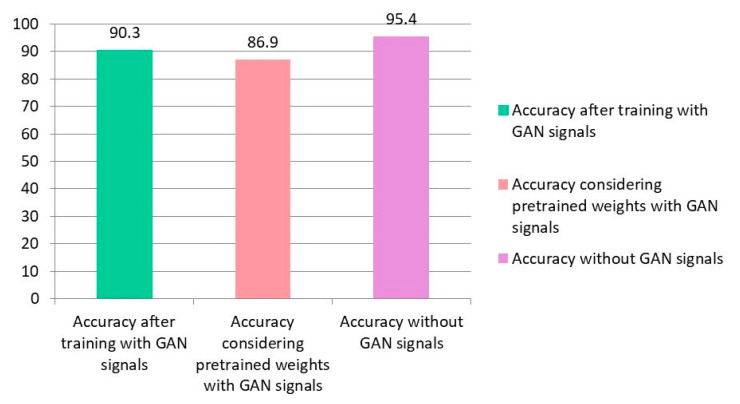
Comparison of computational efficiency of considered networks.

The training of GAN is performed with 9000 iterations. The test accuracy of the proposed CNN with the new dataset after 10-fold cross-validation is equal to 90.3%. The accuracy of the network with the pre-trained weights is performed considering the generated signals, and the obtained test accuracy is equal to 86.9%. Figure 16 confirms the proposed network’s efficiency for classifying the new MindBigData with 9620 14-channel EEG recordings. The details of the layers related to the generative and discriminator subnets are presented in Table 7 and Table 8, respectively.

The evaluation of the proposed CNN-GAN for salient image extraction is accomplished in a 10-fold cross-validation considering the SSIM and CC for each image category of the MNIST dataset. The classification of the EEG database is performed in the first CNN part of the network, and the extracted feature vector is applied as the input to the next GAN deep network to extract the salient image corresponding to the visual stimulation. The results of SSIM and CC are represented in Table 9 and confirm the good performance of the proposed CNN-GAN for reconstructing the salient visual stimulation.

**Figure 17:**
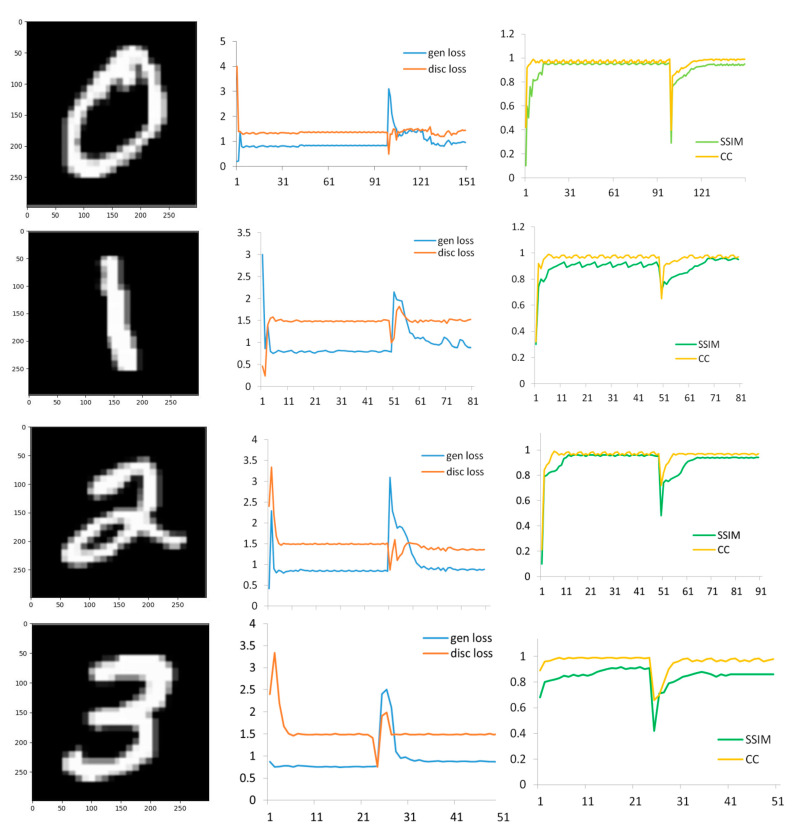
Train accuracy and loss plots of discriminator and generator network for four categories of MNIST dataset; from left to right: MNIST Image, loss trend curves of generator and discriminator for salient image extraction and construction of the original image, CC and SSIM plots for salient image and actual image extraction.

The weights of the trained CNN-GAN for salient image extraction from the brain activity are transferred to the network in order to reconstruct the original image. The initialization of the parameters is performed in the transfer learning procedure, and the adjustment of the new weights to reconstruct the visual stimulation images is accomplished by tracking the loss function of the generator and discriminator networks. The cross-entropy trend curves corresponding to loss function in salient image and original image extraction are illustrated in Figure 17. Furthermore, the plots of tracking the CC and SSIM metrics according to each iteration are represented in this figure considering four categories in the MNIST dataset.

**Figure 18:**
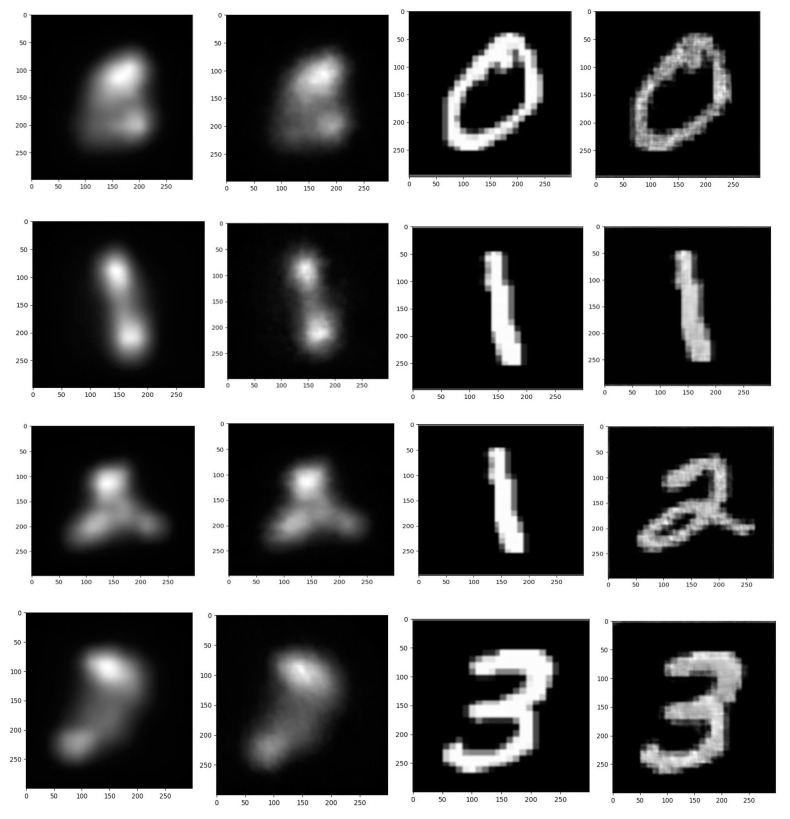
The extracted salient and original images were in four categories, from left to right: the ground reference data, the extracted salient image from brain activity, the original image, and the extracted visual stimulation image using the EEG recordings.

The results of the extracted salient image and reconstructed original image according to four visual stimulation groups are represented in Figure 18. Furthermore, the ground truth image and the actual visual stimulation image are described in this figure. The visual assessment and the evaluation of the SSIM and CC metrics validate the efficiency and good performance of the proposed CNN-GAN framework.

Table 10 compares the performance of the proposed CNN-GAN against other valuable state-of-the-art method methods of SALICON [38], SalNet [39], visual classifier-driven detector [44], neural-driven detector [44], and GNN-based deep network [45] for saliency reconstruction. This table confirms the efficiency of the proposed CNN-GAN method.

One of the restrictions of the proposed method to be overcome in future works is constructing the reference dataset for salient image extraction. This article’s reference data for visual saliency is gathered by implementing the SALICON technique in the CAFFE environment compiled to be compatible with the Python programming language. This would be considered in future works to have salient data using an eye-tracker for tracking the pupil position to identify the visual salient part in the images.

Another recommendation to be considered in future works is more complicated arithmetic content for visual stimulation, and EEG records could be analyzed in these complex situations.

Channel selection is another recommendation to be explored in future works. Studying the effects of different EEG channels in classification and salient arithmetic content extraction would be beneficial. The channels with the most discriminative data could be diagnosed through the experiment.

## 6. Conclusions

This paper proposes an effective convolutional neural network to extract the arithmetic visual stimulation utilizing the brain activity recordings provoked by images of 10 different groups of the MNIST database. The proposed CNN-GAN is trained to extract the salient arithmetic content corresponding to the visual stimulation utilizing the time samples of EEG signal recordings. The trained parameters are used as the initialization weights of the proposed framework to extract the original version of arithmetic visual stimuli images. The application of this research in BCI projects must be addressed. The implementation of the proposed method in this article in the real world would be helpful for disabled or blind subjects to have better interaction with the surrounding environment.

## Figures and Tables

**Table 1:** Channel names of Emotiv EPOC.

Channel Name in Left Hemisphere	Channel Name in Right Hemisphere	Channel Full Name
O1	O2	Occipital
P7	P8	Parietal
T7	T8	Temporal
FC5	FC6	FrontoCentral
F7	F8	Frontal
F3	F4	Frontal
AF3	AF4	Between Prefrontal and Frontal
FP1	FP2	PreFrontal

**Table 2:** Details of the CNN network of the proposed architecture.

Layer Number	Layer Name	Activation Function	Size of Kernel	Strides	Total Number of Weights	Output Size
1	Conv-layer	Leaky ReLU (alpha = 0.1)	5 × 1	1 × 1	14	(1, 14, 14, 250)
2	Normalization					(1, 14, 14, 250)
3	Conv-layer	Leaky ReLU (alpha = 0.1)	5 × 1	1 × 1	10	(1, 10, 14, 250)
4	Normalization					(1, 10, 14, 250)
5	Conv-layer	Leaky ReLU (alpha = 0.1)	5 × 1	1 × 1	10	(1, 10, 14, 250)
6	Normalization					(1, 10, 14, 250)
7	Full-connected					(1, 3500)
8	Full-connected					(1, 2500)

**Table 3:** The details of the layers in the generator of the proposed CNN-GAN.

Layer Number	Layer Name	Activation Function	Size of Kernel	Number of Kernels	Strides	Total Number of Weights	Output of the Layer
1	Full-connected					250,000	(1, 100)
2	Full-connected	Rectified LU (0.1)				2,000,000	(1, 20,000)
3	Reshape					0	(1, 50, 50, 8)
4	Conv 2-D Transposed	Rectified LU (0.1)	4 × 4	6	2 × 2	768	(1, 100, 100, 6)
5	Conv 2-D Transposed	Rectified LU (0.1)	4 × 4	6	3 × 3	768	(1, 300, 300, 6)
6	Conv 2-D Transposed	Rectified LU (0.1)	4 × 4	6	1 × 1	768	(1, 300, 300, 6)
7	Conv 2-D Transposed	Rectified LU (0.1)	4 × 4	6	1 × 1	768	(1, 300, 300, 6)
8	Conv 2-D	Rectified LU (0.1)	2 × 2	1	2 × 2	33	(1, 299, 299, 1)

**Table 4:** The details of the layers in the discriminator of the proposed CNN-GAN.

Layer	Layer Name	Activation Function	Size of Kernel	Kernels	Stride	Total Number of Weights	Output Weight
1	Conv 2-D	Rectified LU (0.1)	4	2	2	32	(None, 150, 150, 2)
2	Dropout (rate = 0.2)					0	(None, 150, 150, 2)
3	Conv 2-D	Rectified LU (0.1)	4	2	2	130	(None, 75, 75, 2)
4	Dropout (rate = 0.2)					0	(None, 75, 75, 2)
5	Conv 2-D	Rectified LU (0.1)	4	2	2	130	(None, 38, 38, 2)
6	Dropout (rate = 0.2)					0	(None, 38, 38, 2)
7	Flattening					0	(1, 2888)
8	Full-connected					2889	(1, 1)

**Table 5:** Search scope and optimal values in the training procedure.

Parameters	Search Scope	Optimal Value
Optimizer for CNN	SGD, Adam	SGD
Loss-function	Cross-Entropy, MSE	Cross-Entropy
Number of convolutional layers	1, 2, 3, 4	3
Learning-rate for CNN	0.001, 0.01, 0.1	0.001
Weight loss of SGD for CNN part	5 × 10^−5^, 5 × 10^−3^	5 × 10^−5^
Dropout rate of CNN	0.2, 0.3	0.2
Optimizer for GAN	SGD, Adam	Adam
Learning-rate for GAN	0.01, 0.001, 0.0001, 0.00001	0.0001
Number of 2D-conv transposed layers of generator	4, 3, 2	4
Number of 2D-conv layers of discriminator	4, 3, 2	3
Filters for the first conv-layer in CNN	10, 14, 28	14
Filters for the second conv-layer in CNN	10, 14, 30	10

**Table 6:** Comparison of different methods for classification of the MNIST-EEG.

Evaluation Metrics	CNN	CNN + LSTM	GNN [46]	LSTM [14]
Accuracy	95.4%	86.7%	73%	84.3%
Precision	96.7%	87.8%	73.6%	84.52%
F1-score	96.7%	87.8%	73.6%	84.52%
Cohen’s Kappa Coefficient	96.7%	87.8%	73.6%	84.52%

**Table 7:** The details of layers in the generator of GAN.

Layer Number	Layer Name	Activation Function	Ouput Size	Size of Kernel	Strides	Number of Kernels	Padding
1	Full-Connected	-	(7 × 125 × 8)				
2	Conv-2D Transposed	ReLU (alpha = 0.3)	(7, 125, 8)	1 × 4	1 × 1	8	Yes
3	Conv-2D Transposed	ReLU (alpha = 0.3)	(7, 125, 8)	1 × 4	1 × 1	8	Yes
4	Conv-2D Transposed	ReLU (alpha = 0.3)	(14, 250, 30)	1 × 4	2 × 2	30	Yes

**Table 8:** The details of layers in the discriminator of GAN.

Layer Number	Layer Name	Activation Function	Output Size	Size of Kernel	Strides	Number of Kernels	Padding
1	Conv-2D	ReLU (alpha = 0.3)	(1, 14, 250, 6)	1 × 4	1 × 1	6	Yes
2	Dropout (0.2)	-	(1, 14, 250, 6)				
3	Conv-2D	ReLU (alpha = 0.3)	(1, 7, 125, 6)	1 × 4	2 × 2	6	Yes
4	Dropout (0.2)	-	(1, 7, 125, 6)				
5	Conv-2D	ReLU (alpha = 0.3)	(1, 7, 125, 6)	1 × 4	1 × 1	6	Yes
6	Dropout (0.2)	-	(1, 7, 125, 6)				
7	Flatten	-	(1, 5250)				
8	Fully Connected	-	(1, 1)				

**Table 9:** The saliency validation metrics for visual salient reconstruction.

Category Number	Arithmetic Category	SSIM	CC
1	Zero	91.7	95.6
2	One	93.2	98.2
3	Two	95.3	97.1
4	Three	92.4	96.8
5	Four	91.5	96.1
6	Five	91.1	96.8
7	Six	94.8	99.4
8	Seven	93.6	97.7
9	Eight	94.5	99.2
10	Nine	91.8	95.9
-	Average	92.9	97.28

**Table 10:** Comparison of different methods for saliency detection.

Method	Dataset	SSIM	CC
Visual classifier-driven detector [44]	EEG-ImageNet	-	17.30%
Neural-driven detector [44]	EEG-ImageNet	-	35.7%
SalNet [39]	ImageNet	-	27.10%
SALICON [38]	ImageNet	-	34.8%
GNN-based deep network [45]	EEG-ImageNet	89.46%	99.39%
CNN-GAN	MindBigData	92.9%	97.28%

## Data Availability

The EEG dataset is available online at https://mindbigdata.com/opendb/ (Accessed on 12 February 2020).

## Nomenclature

BCI	Brain–Computer Interface
CNN	Convolutional Neural Network
EEG	Electroencephalogram
FN	False Negative
FP	False Positive
GAN	Generative Adversarial Network
GNN	Graph Neural Network
LSTM	Long Short-Term Memory
MNIST	Modified National Institute of Standards and Technology
SALIENCY	Saliency in Context
SGD	Standard Gradient Descent
SIM	Similarity
SSIM	Structural Similarity
SSVEP	Steady-State Visually Evoked Potential
SVM	Support Vector Machine
TN	True Negative
TP	True Positive
VGG	Visual Geometry Group
